# Gut Microbiome and Lipidome Signatures in Irritable Bowel Syndrome Patients from a Low-Income, Food-Desert Area: A Pilot Study

**DOI:** 10.3390/microorganisms11102503

**Published:** 2023-10-06

**Authors:** Nikita Paripati, Lauren Nesi, John D. Sterrett, Lamya’a M. Dawud, Lyanna R. Kessler, Christopher A. Lowry, Lark J. Perez, Joshua DeSipio, Sangita Phadtare

**Affiliations:** 1 Department of Biomedical Sciences, Cooper Medical School of Rowan University, Camden, NJ 08103, USA; nikita.paripati@pennmedicine.upenn.edu (N.P.); lnesi@dmc.org (L.N.); desipio-joshua@cooperhealth.edu (J.D.); 2 Department of Emergency Medicine, Penn Medicine, Pittsburgh, PA 15261, USA; 3 Department of Urology, Detroit Medical Center, Detroit, MI 4820, USA; 4 Department of Integrative Physiology, University of Colorado Boulder, Boulder, CO 80309, USA; john.sterrett@colorado.edu (J.D.S.); lamyaa.dawud@colorado.edu (L.M.D.); lyanna.kessler@colorado.edu (L.R.K.); christopher.lowry@colorado.edu (C.A.L.); 5 Department of Chemistry and Biochemistry, Rowan University, Glassboro, NJ 08028, USA; perezla@rowan.edu; 6 Department of Gastroenterology, Cooper University Hospital, Camden, NJ 08103, USA

**Keywords:** gut microbiome, lipidome, irritable bowel syndrome, dysbiosis, high-fructose corn syrup

## Abstract

Irritable bowel syndrome (IBS) is a common gastroenterological disorder with triggers such as fructose. We showed that our IBS patients suffering from socioeconomic challenges have a significantly high consumption of high-fructose corn syrup (HFCS). Here, we characterize gut microbial dysbiosis and fatty acid changes, with respect to IBS, HFCS consumption, and socioeconomic factors. Fecal samples from IBS patients and healthy controls were subjected to microbiome and lipidome analyses. We assessed phylogenetic diversity and community composition of the microbiomes, and used linear discriminant analysis effect size (LEfSe), analysis of compositions of microbiomes (ANCOM) on highly co-occurring subcommunities (modules), least absolute shrinkage and selection operator (LASSO) on phylogenetic isometric log-ratio transformed (PhILR) taxon abundances to identify differentially abundant taxa. Based on a Procrustes randomization test, the microbiome and lipidome datasets correlated significantly (*p* = 0.002). Alpha diversity correlated with economic factors (*p* < 0.001). Multiple subsets of the phylogenetic tree were associated with HFCS consumption (*p* < 0.001). In IBS patients, relative abundances of potentially beneficial bacteria such as Monoglobaceae, Lachnospiraceae, and Ruminococcaceae were lower (*p* = 0.007), and *Eisenbergiella*, associated with inflammatory disorders, was higher. In IBS patients, certain saturated fatty acids were higher and unsaturated fatty acids were lower (*p* < 0.05). Our study aims first to underscore the influence of HFCS consumption and socioeconomic factors on IBS pathophysiology, and provides new insights that inform patient care.

## 1. Introduction

The gastrointestinal tract along with the microbiome that inhabits it is a complex organ system that has functions beyond digestion and absorption such as immunological and neurological functions [1]. The dysregulation of these functions leads to the development of several digestive disorders [2]. One of the most common of these disorders is irritable bowel syndrome (IBS) [3]. Common symptoms of IBS include abdominal pain, bloating, and altered bowel habits. It is one of the most common gastroenterological disorders, affecting 10–15% of the global population with varied regional prevalence. Direct and indirect costs of IBS in the United States result in healthcare costs of more than USD 30 billion/year [4,5]. IBS is considered a debilitating disorder as it significantly affects the quality of life of patients. The precise etiology and pathophysiology of IBS are yet to be resolved; however, altered gut motility, visceral hypersensitivity, inflammation, altered gut microbiome, psychiatric disorders, and neurological disturbances are frequently associated with IBS [6]. The complex etiology, multifactorial pathophysiology, involvement of psychological comorbidities, heterogeneity of clinical manifestations, and lack of effective diagnostic methods for IBS pose difficulty in the development of more efficacious treatment strategies [7]. There are now increasing numbers of reports that the COVID-19 pandemic has further worsened the gastrointestinal and psychological symptoms of IBS patients [8,9].

Food triggers have been reported for IBS. Among the possible associated causes of IBS are fructose intolerance and an elevated consumption of fructose products. Up to one third of patients with IBS may have dietary fructose intolerance or fructose malabsorption [10,11,12]. High-fructose corn syrup (HFCS) is a major source of fructose in American diets, representing up to 40% of caloric sweeteners added to foods and beverages [13]. Recently, we reported a significantly high consumption of HFCS in our IBS patients. These patients suffer from socioeconomic challenges [14]. Our hospital is located in Camden, New Jersey. It is estimated that 37.4% of Camden residents live below the poverty line, with a median household income of USD 26,105. This is less than half of the median household income in the United States [15]. Other highlights of this community include high unemployment rate, low education levels, and “food desert” status [16]. The lack of access to nutritious and affordable food items leads to limited dietary choices for the residents. Readily available HFCS-containing inexpensive food products thus become staple foods.

Studies have been carried out to elucidate the gut microbiome profile of IBS compared to that of healthy control participants [17,18,19]. Kim et al. carried out a case-control study and a cross-cohort analysis of gut dysbiosis in IBS [20]. A meta-analysis by Pittayanon et al. reported a higher prevalence of Firmicutes and a lower prevalence of Bacteroidetes in IBS patients [21]. Wang et al. carried out a systematic review and meta-analysis of 6333 case-control studies of gut microbiome dysbiosis in IBS [22]. Some studies have focused on the brain–gut axis aspect of the disease (i.e., signaling from the brain to the gut) [23,24,25]. As dietary modification is one of the major methods of management for IBS patients, a few groups have also studied the effect of certain diets on IBS. A study showed that interventions such as a low-FODMAP (fermentable oligosaccharides, disaccharides, monosaccharides, and polyols) diet for IBS patients altered their gut microbiome’s taxonomy and functional potential to more closely resemble healthy controls, and that IBS patients with “pathogenic” baseline microbiomes had a greater reduction in symptoms from the low-FODMAP diet than IBS patients with healthy-like microbiomes [26]. Another study analyzed the gut microbiomes of IBS patients on exclusion (including gluten-free, dairy-free, or low-FODMAP) diets. Vandeputte and Joossens discussed the effects of low- and high-FODMAP diets on gut microbiome composition with respect to GI diseases [27]. Van Lanen et al. carried out a systematic review and meta-analysis of the efficacy of a low-FODMAP diet in adult IBS [28].

This study was undertaken to characterize the gut microbiome and changes in fatty acid concentrations with respect to IBS phenotypes. These changes were then evaluated with respect to a high consumption of HFCS-containing foods in our IBS patient population as well as other dietary, lifestyle, and socioeconomic factors such as alcohol consumption, household income, persons per household, etc., that may influence the manifestation and progression of IBS. To the best of our knowledge, this is the first study to underscore the influence of HFCS consumption and socioeconomic factors in IBS pathophysiology based on distinct microbiome and lipidome differences. These correlations are relevant from a clinical standpoint, especially when helping underserved communities. This study aims to help guide further research and improve IBS patient care regarding dietary interventions to avoid the exacerbation of gastrointestinal and coexisting psychological comorbidities.

## 2. Materials and Methods

### 2.1. Patients and Sample Collection

Our study included patients that presented to the Cooper University Hospital (CUH) in Camden, New Jersey. IBS patients at least 18 years of age with ongoing care at CUH were eligible for this study. Exclusion criteria included age <18 years, non-English-speaking patients, pregnancy, and antibiotics in last three months. IBS patients are identified in our electronic medical record system (EPIC) with ICD-10 codes of K58.0, K58.1, K58.2, K58.8, and K58.9. ROME IV criteria were used to determine IBS subtypes as: (i) IBS with constipation (IBS-C); (ii) IBS with diarrhea (IBS-D); (iii) mixed IBS (IBS-M), or (iv) unclassified IBS [29,30]. Control participants were recruited from a healthy population from the same area, who reported to CUH for wellness visits. Screening was carried out to confirm that the control subjects did not have a previous history or current documentation of IBS. They also did not have any GI-related symptoms before recruitment. Informed consent was obtained as per the IRB guidelines. We reviewed the medical records of the participants to confirm demographic and clinical details. Surveys were also collected from the participants regarding their demographic information and dietary history. We have described these surveys previously [14]. In brief, the survey included questions about demographic variables (sex, gender, ethnicity/race), lifestyle factors (residence, education level, marital status, household income, persons per household, alcohol, and tobacco use), dietary factors (consumption of HFCS-rich foods), and clinical data (height, weight, type of IBS, colonoscopy/endoscopy, medications, hypertension, diabetes, cholesterol, and psychological comorbidities). A total of 398 food items listed as high in HFCS were included in the surveys; these were identified using the United States Department of Agriculture (USDA) database. Patients were asked to provide a stool sample. Samples were collected in Norgen Stool Nucleic Acid Collection and Preservation Tubes (Norgen Biotek Corp., Thorold, ON, Canada) and were frozen at −80 °C as described previously [31,32,33].

### 2.2. 16S rRNA Gene Sequencing

Genomic bacterial DNA collected from fecal samples was extracted using a Qiagen DNeasy PowerSoil HTP extraction kit (Qiagen, Redwood City, CA, USA). Manufacturer’s instructions were followed for the procedure. The marker genes in the extracted DNA were amplified via PCR using GoTaq Master Mix (Promega, Fitchburg, WI, USA). High-throughput sequencing was conducted using the Golay barcode primers 515 F (5′-GTGCCAGCMGCCGCGGTAA-3′) and 806 R (5′-GGACTACHVGGGTWTCTAAT-3′), respectively. The Golay barcode primers target the V4 hypervariable region of the 16S rRNA gene, which has been found to be highly conserved and useful for the taxonomic profiling of the gut microbiome [34]. PCR amplification methods include heating at 94 °C for 3 min followed by 35 × 94 °C for 45 s, 55 °C for 1 min, and 72 °C for 1.5 min, with the final extension being carried out at 72 °C for 10 min. To purify and normalize the PCR products, the SequelPrep Normalization Kit (Cat. No. A1051001, ThermoFisher, Waltham, MA, USA) was used. Library preparation and gene sequencing for the 16S rRNA gene were carried out using the V2 300-cycle Illumina MiSeq System.

### 2.3. Analysis of Lipids

Stool samples were homogenized, and aliquots (100 mg) were analyzed via gas chromatography–mass spectrometry (GC-MS). Fatty acids were extracted through liquid/liquid extraction, which also removes the nucleic acid preservative. An aliquot (250 μL) of each extract was transferred to a fresh analysis tube. Removal of the solvent was carried out through evaporation under a stream of nitrogen. Internal standard solution was added to the tubes that contained dried sample extracts, quality controls (QCs), and calibration standards. Evaporation under nitrogen was used to remove the solvent. Processing of the dried samples and QCs by methylation/transmethylation with methanol/sulfuric acid resulted in the formation of the corresponding fatty acid methyl esters (FAME) of free fatty acids and conjugated fatty acids. The reaction mixture was neutralized and extracted with hexane, which was then analyzed using 7890A/5975C GC/MS in the single-ion monitoring (SIM) positive mode with electron ionization. Linear and quadratic regression analyses were generated from fortified calibration standards prepared immediately prior to each run. Quantitation was performed using both these analyses. Agilent MassHunter GC/MS Acquisition B.07.04.2260 and Agilent MassHunter Workstation Software Quantitative Analysis for GC/MS B.09.00/Build 9.0.647.0 were used for collection and processing of raw data. The total fecal content of 30 fatty acids was measured after conversion into their corresponding FAMEs. Concentrations are provided in weight-corrected μg/g of fecal dry mass. Values below the lower limit of quantification were treated as a concentration of 0.001 μg/mL.

### 2.4. Microbiome Data Processing and Statistical Analysis

Data generated through 16S rRNA gene sequencing were analyzed using Quantitative Insights Into Microbial Ecology (QIIME) 2 2020.11 as well as Python-based packages (Python 3.6.12) with packages in accordance with the QIIME 2 2020.11 environment. Sequences were de-multiplexed, filtered, and clustered into amplicon sequence variants (ASVs) using QIIME 2 DADA2 [35]. The phylogenetic tree was created using SATé-enabled phylogenetic placement (SEPP) via QIIME 2 [36]. A naïve Bayes classifier trained on the latest SILVA version 138 16S rRNA gene database (March 2021) was used to assign the taxonomy via the QIIME 2 interface. Additional Python packages (SciPy, Statsmodels, Scikit-bio) were used for statistical tests on QIIME 2-generated data. Diversity analyses were performed in QIIME 2, rarefied to an even sampling depth of 54,000 reads per sample [37]. Faith’s phylogenetic diversity (Faith’s PD) was considered the primary metric for microbiome richness, and unweighted UniFrac was considered the primary metric for beta diversity. PERMANOVA was used to assess group-based differences in microbiome community composition (beta diversity). Stacked bar plots were created using the R package microshades v1.10 [38].

### 2.5. Procrustes Randomization Test

Principal coordinate analysis (PCoA, using the Python package Scikit-learn) was performed on Bray–Curtis distance matrices from both the fecal lipidome and fecal microbiome datasets (using the Python package SciPy.distance), and a Procrustes test was performed on the lipidome and microbiome PCoA coordinates. Following the protocol from Peres-Neto and Jackson, a Procrustes randomization test (PROTEST) was performed by randomly permuting the PCoA coordinates and performing a Procrustes test on the permuted samples 10,000 times. The *p* value was calculated based on the portion of randomized Procrustes tests with resulting *m*^2^ (Gower’s statistic) scores lower than the Procrustes *m*^2^ score for the observed datasets [39].

### 2.6. Differential Abundance Testing

To identify differentially abundant taxa based on IBS status and subtype, we used Analysis of Compositions of Microbiomes (ANCOM, via Scikit-bio) [40]. Further, we also created a co-occurrence network of taxa and lipids, from which we grouped features into highly correlated modules, which were summed for analysis to identify differential groups of features. Specifically, we filtered rare taxa with total read counts <500 and average read counts <3 [41]. Taxa were center log-ratio transformed to account for compositionality, while lipids were untransformed. Pearson’s correlation coefficients were calculated pairwise between all features. Edges were drawn only between features with a Pearson’s *rho* > 0.5 and *p* < 0.05. The Louvain modularity maximization algorithm was applied to identify modules with many strong connections within modules and few connections between modules [42]. Relative abundances of all features in each module were summed, and IBS-based differences in module abundances were assessed via a Kruskal–Wallis test.

Next, we utilized a phylogenetic isometric log-ratio transformation (PhILR) to identify taxa associated with HFCS consumption to test our assumption that HFCS consumption causes a phylogenetic shift in the gut microbiome. The PhILR transformation considers ratios between subtrees of the phylogenetic tree to use phylogeny to transform compositional microbiome data outside the simplex, such that each feature can change independently of the others [43]. At each branching point in the tree, the isometric log-ratio of the summed read counts in one subtree of the branch to the other subtree was calculated to create our “balances”. Least absolute shrinkage and selection operator (LASSO) regression, with lambda (penalty parameter) = 3000, was used to identify the top 5 balances associated with HFCS consumption. In R, PhILR was performed using the philr package v1.24.0, LASSO was performed using the glmnet package v4.7, and balances were visualized using ggtree v3.6.2 [44,45].

### 2.7. Ethics Approval

This study received approval from the Cooper Health System Institutional Review Board (IRB) (17-079EX), and all the steps were carried out as per the standards set by the IRB. All procedures performed in studies involving human participants were in accordance with the ethical standards of the institutional and/or national research committee and with the 1964 Helsinki Declaration and its later amendments or comparable ethical standards.

## 3. Results

### 3.1. Patient Demographics

The demographic data for the study participants, collected via surveys and confirmed by chart review, are presented in Table 1. Consistent with the literature, refs. [10,46,47,48], a majority of the IBS patients that participated in this study were females (78.8%), >40 years of age (63.6%), and Caucasian (84.8%). Most IBS patients (96.7%) did not smoke. A majority of the IBS patients did not have diabetes, high cholesterol, or hypertension. Psychological comorbidities such as depression (12 patients; 36%) and anxiety (13 patients; 39%) were noted in IBS patients. Control subjects did not have depression or anxiety. All participants in this study were included for all analyses; no outliers were removed from any statistical testing or figures.

### 3.2. Gut Microbiome Analysis

There were no differences in the richness of the gut microbiome between IBS patients and controls or across IBS subtypes, as assessed via a Kruskal–Wallis test on the Faith’s phylogenetic diversity of each sample (*H*(3) = 4.1, *p* = 0.25). IBS patients had a mean Faith’s phylogenetic diversity of 48.7 (95% confidence interval 44.9–52.5), whereas healthy control participants had a mean of 47.8 (95% confidence interval 42.8–52.8).

#### 3.2.1. Association of the Community Composition of the Gut Microbiome with IBS Group

**Figure 1:**
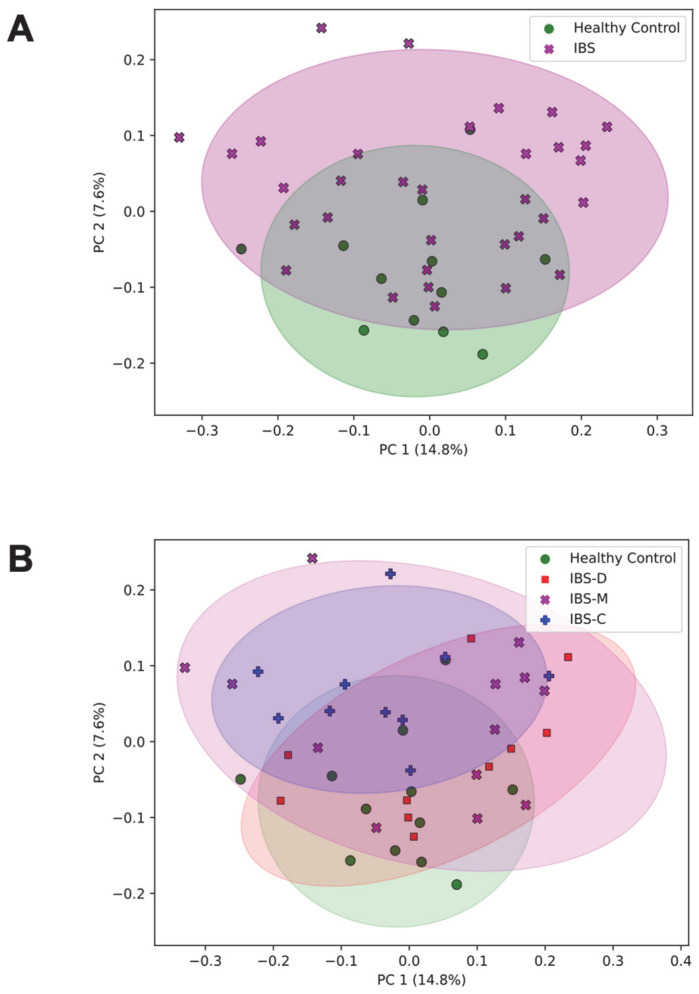
Microbiome beta diversity of healthy controls versus IBS and IBS subtypes. (**A**) Unweighted UniFrac PCoA plots are shown comparing healthy control participants (green, *n* = 12) and IBS patient samples (purple, *n* = 33) and between (**B**) healthy control participants (green, *n* = 12) and IBS subtypes: IBS-D (red, *n* = 10), IBS-M (purple, *n* = 13), and IBS-C (blue, *n* = 10). Percentages along each axis show the portion of phylogenetic variance across samples captured by that axis. Each point represents the phylogenetic composition of one sample. Ellipses represent 95% confidence intervals of the group’s PCoA coordinates. Abbreviations: IBS, irritable bowel syndrome; IBS-D, irritable bowel syndrome–diarrhea; IBS-M, irritable bowel syndrome-mixed; IBS-C, irritable bowel syndrome–constipation, principal coordinates analysis axis (PC), principal coordinates analysis (PCoA).

**Figure 2:**
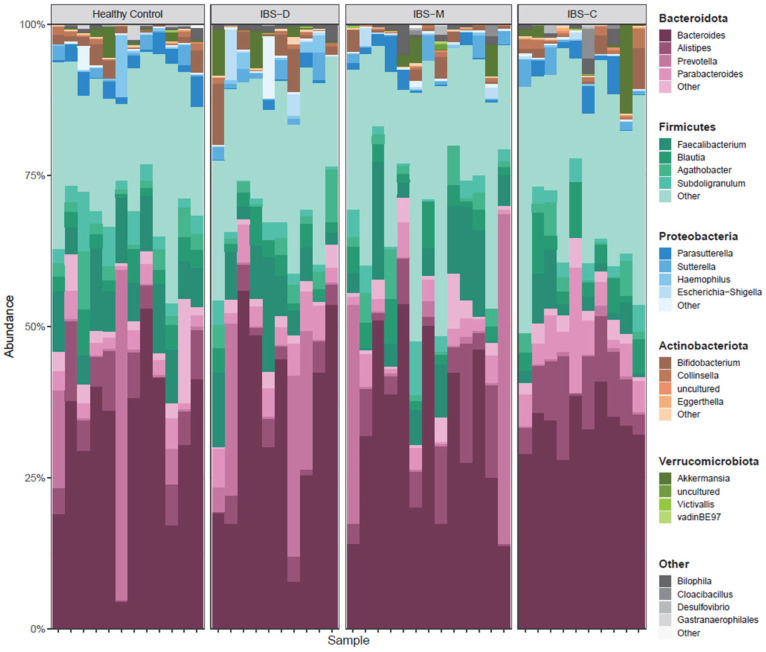
Taxa bar plot of healthy controls versus IBS and IBS subtypes. Taxa bar plot shows relative abundances of phyla in each sample. Each vertical bar represents one sample, and the value on the y-axis represents the relative abundance of each taxon (separated by color) in that sample. Names of taxa corresponding to taxa are shown to the right of the plot, with bold headers indicating the phylum-level and non-bold text indicating the genus-level taxonomic assignment. Abbreviations: IBS, irritable bowel syndrome; IBS-D, irritable bowel syndrome–diarrhea; IBS-M, irritable bowel syndrome–mixed; IBS-C, irritable bowel syndrome–constipation.

Unweighted UniFrac principal coordinate analysis (PCoA) (Figure 1) shows the ordination of the healthy control participants and IBS patients. PCoA plots are shown comparing healthy control participants and IBS patient samples (Figure 1A) and healthy control subjects and IBS subtypes (Figure 1B). Each point represents the phylogenetic composition of one sample, and shaded regions represent 95% confidence intervals of the first two PCoA axes for each group. Points that are close together have similar phylogenetic composition, and points that are far apart have dissimilar phylogenetic composition. The proportion of variance explained by each principal coordinate axis is denoted in the corresponding axis label; PC1 explains 14.8% of the variation and PC2 explains 7.6% of the variation across samples. IBS patients had significantly different overall community composition, compared to control participants, based on unweighted UniFrac PERMANOVA (pseudo-*F* = 1.63, *p* = 0.021; Figure 1A). Additionally, there were significant differences in microbiome community composition across healthy controls and IBS subtype (pseudo-*F* = 1.21, *p* = 0.020; Figure 1B). A stacked bar plot in Figure 2 shows the taxonomic composition of the healthy control and IBS subtype samples. Bacteroidota and Firmicutes were predominant in all groups of samples.

#### 3.2.2. Association of Differentially Abundant Gut Microbiome Taxa with IBS Groups

**Figure 3:**
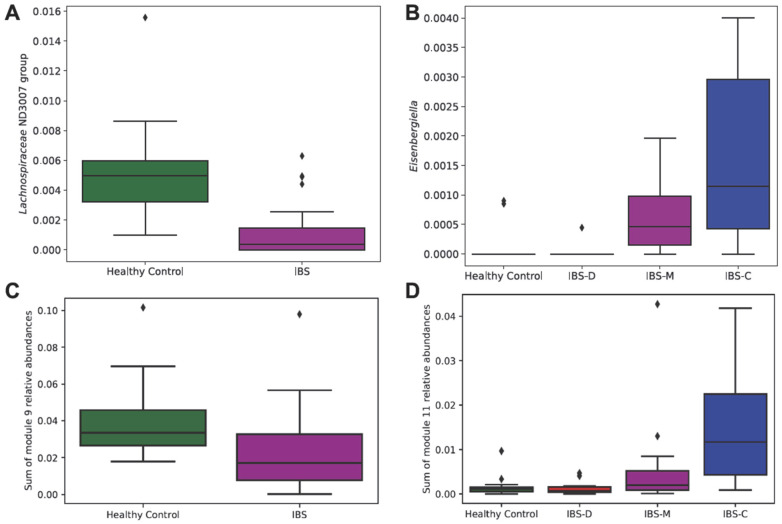
Differentially abundant taxa in IBS and IBS subtypes versus healthy controls. Taxonomic differences in the microbiomes of healthy control participants and IBS patients were analyzed via ANCOM analysis (**A**,**B**) and module network analysis (**C**,**D**). ANCOM analyses of healthy control participants versus IBS patients (**A**) and of healthy control participants versus IBS subtypes (IBS-D, IBS-M, and IBS-C) (**B**) are shown. The summed relative abundances of modules (module 9; (**C**) and module 11; (**D**) identified from co-occurrence networks within our dataset are shown in the lower panel. Abbreviations: ANCOM, analysis of compositions of microbiomes; IBS, irritable bowel syndrome; IBS-D, irritable bowel syndrome–diarrhea; IBS-M, irritable bowel syndrome–mixed; IBS-C, irritable bowel syndrome–constipation.

Next, we assessed the differences in the specific taxa (ANCOM) and groups of taxa (Kruskal–Wallis on summed network modules) in the microbiomes of the healthy control participants versus the IBS patients. The relative abundance of *Lachnospiraceae* group ND3007, which ANCOM identified as differentially abundant (*W* = 42/254), was lower in participants with IBS than healthy controls (Figure 3A). Additionally, the relative abundance of *Eisenbergiella*, which ANCOM identified as differentially abundant (*W* = 150/254), was higher in participants with IBS-C and IBS-M, relative to healthy controls and participants with IBS-D (Figure 3B). ANCOM is a conservative but robust method using pairwise log-ratios to account for the compositionality of the data; thus, the outcomes usually include only few differentially abundant taxa.

The network module analysis (Louvain modularity maximization) identified eleven modules, or highly co-occurring subcommunities, of bacteria; the detailed composition of modules is presented in Supplementary Table S1. The summed relative abundances of two modules within our dataset were different based on IBS status (Figure 3C,D). The relative abundance of module 9, which contained members of the class Clostridia (including the families Monoglobacea, Lachnospiraceae, and Ruminococcaceae), was lower in participants with IBS, relative to healthy controls (*H*(1) = 7.1, *p* = 0.007) (Figure 3C). The relative abundance of module 11, which contained different members of the class Clostridia, was highest in participants with IBS-C (*H*(3) *=* 13.7, *p* = 0.003) (Figure 3D).

**Figure 4:**
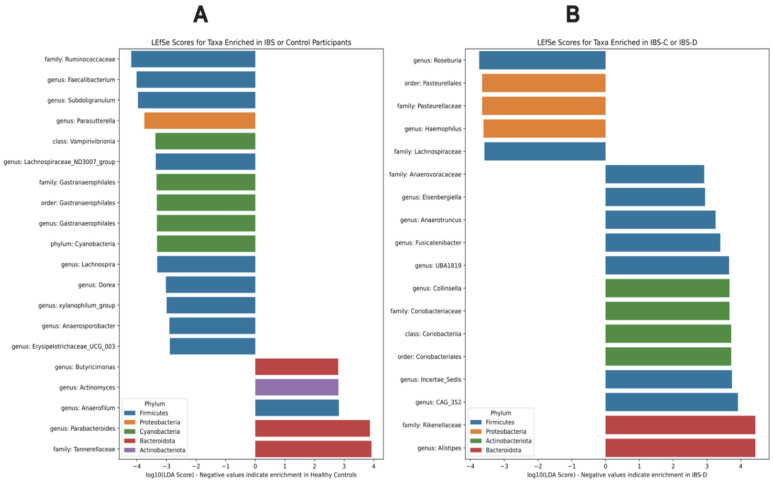
Linear discriminant analysis effect size (LEfSe) scores for taxa enriched in healthy control participants or IBS patients (**A**) and IBS-C or IBS-D subtypes (**B**). Negative values in (**A**) represent taxa that were enriched in healthy control participants, whereas positive values represent taxa that were enriched in IBS patients. Negative values in (**B**) represent taxa that were enriched in IBS-D patients, whereas positive values represent taxa that were enriched in IBS-C patients. Color represents the phylum. Abbreviations: IBS, irritable bowel syndrome; IBS-D, irritable bowel syndrome–diarrhea; IBS-M, irritable bowel syndrome–mixed; IBS-C, irritable bowel syndrome–constipation;LEfSe, linear discriminant analysis effect size.

Next, we carried out linear discriminant analysis effect size (LEfSe) analysis. LEfSe scores for taxa enriched in healthy control participants or IBS patients are shown in Figure 4A, whereas LEfSe scores for taxa enriched in IBS-C versus IBS-D subtypes are shown in Figure 4B. As seen from Figure 4A, purportedly beneficial bacteria belonging to Ruminococcaceae were highest in the healthy control participants, while the genera *Butyricimonas* and *Parabacteroides* and the family Tannerellaceae within the Bacteroidota phylum were enriched in IBS patients as compared to healthy control participants (Figure 4A). The relative abundances of the genus *Alistipes* and family Rikenellaceae within the Bacteroidota phylum were higher in IBS-C patients than in IBS-D patients (Figure 4B).

### 3.3. Monthly HFCS Consumption with Respect to IBS Groups

**Figure 5:**
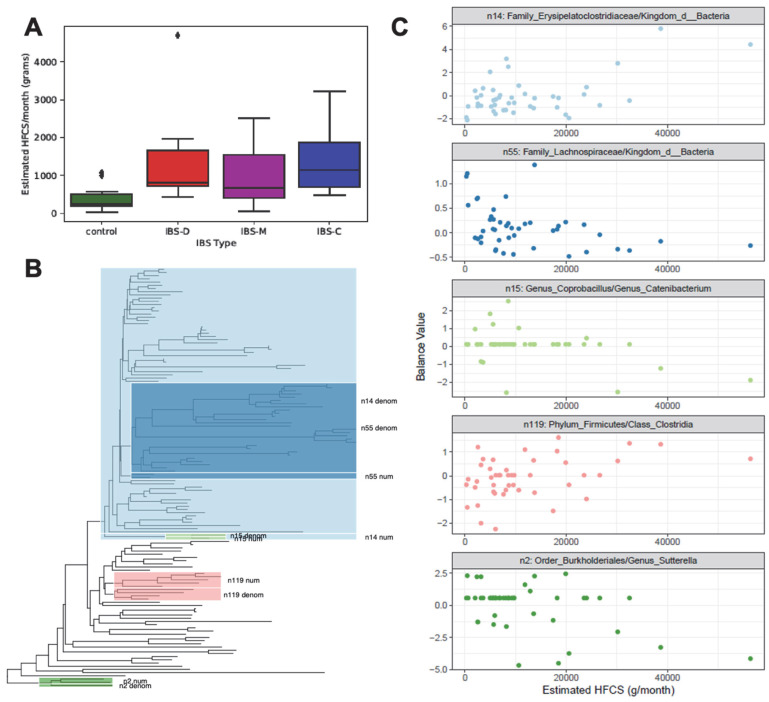
HFCS consumption (g/per month) of healthy control participants and IBS subtypes (**A**) and phylogenetic isometric log-ratio transformation (PhILR) analysis of taxa associated with HFCS consumption (**B**,**C**). (**A**) Boxplots show estimated HFCS consumption, separated into IBS subtypes. Horizontal lines represent the median. Boxes indicate the 1st through 3rd quartiles of monthly HFCS consumption in each IBS subtype, and whiskers represent the range up to 1.5× the interquartile range. Diamonds represent monthly consumption for an individual outside of 1.5× the interquartile range. (**B**) A phylogenetic tree constructed using 16S amplicon sequence variants is highlighted to show PhILR balances associated with HFCS consumption. Balances were constructed at each branching point in the phylogenetic tree, using the isometric log-ratio of one subtree to the other. As such, the numerator and denominator of each balance are signified using “num” and “denom”. Balances shown were associated with HFCS consumption, per LASSO regression. (**C**) The relationship between phylogenetic balances associated with HFCS consumption are shown using a scatter plot, where each point depicts that balance’s value on the y-axis as a function of HFCS consumption on the x-axis, and each point represents one participant’s sample, and samples from all participants were included. Abbreviations: IBS, irritable bowel syndrome; IBS-D, irritable bowel syndrome–diarrhea; IBS-M, irritable bowel syndrome–mixed; IBS-C, irritable bowel syndrome–constipation; LASSO, least absolute squares shrinkage and selection operator; PhILR, phylogenetic isometric log-ratio transformation.

Previously, we observed a higher consumption of HFCS-containing food items in our IBS patient population [14]. Consistent with that observation, a higher estimated consumption of HFCS in all IBS subtypes relative to control participants was also observed in the present study using ANOVA followed by Dunn’s post hoc tests (*F*(3,41) = 3.4, *p* = 0.026, Figure 5A). The participants in the current study are different to those mentioned in the previous study. All IBS subtypes had significantly higher estimated HFCS consumption than the controls. No significant difference was observed among the IBS subtypes.

### 3.4. Phylogenetic Groups of Taxa in the Gut Microbiome with Respect to Monthly HFCS Consumption

As diet can influence the growth of microorganisms, it can be hypothesized that a higher consumption of HFCS will cause a phylogenetic shift in the gut microbiome. Thus, we analyzed phylogenetic isometric log-ratio transformed (PhILR) abundances to identify taxa associated with HFCS consumption (Figure 5B,C). LASSO regression on PhILR-transformed microbiome data identified five phylogenetic balances of taxa that were associated with estimated HFCS consumption (adjusted *R*^2^ = 0.388, *F*(5,39) = 6.6, *p* < 0.001). One of the most strongly positively associated phylogenetic balances was the ratio of the family Erysipelatoclostridiaceae to a broad section of the phylogenetic tree predominantly including the classes Clostridia and Bacteroidia (balance n14). Additionally, HFCS was negatively associated with a ratio between members of the family Lachnospiraceae (*Eubacterium ventriosum* and an ASV within the genus *Anaerosporobacter*) and a broad section of the phylogenetic tree predominantly including the classes Clostridia and Bacteroidia (n55). Within the Erysipelatoclostridiaceae family, we identified a negative association between estimated HFCS consumption and the ratio of the genera *Coprobacillus* to *Catenibacterium* (balance n15). The ratio of taxa in the phylum Firmicutes and class Clostridia was positively associated with estimated HFCS consumption (n119). Finally, we identified a negative association between estimated HFCS consumption and a balance within the order Burkholderiales, which was the ratio of two taxa (one *Sutterella* ASV and one within the family Oxalobacteraceae) to another *Sutterella* ASV, supporting that within-genus (and potentially strain-level) differences are associated with dietary patterns.

### 3.5. Association of Fecal Lipids with IBS Groups

**Figure 6:**
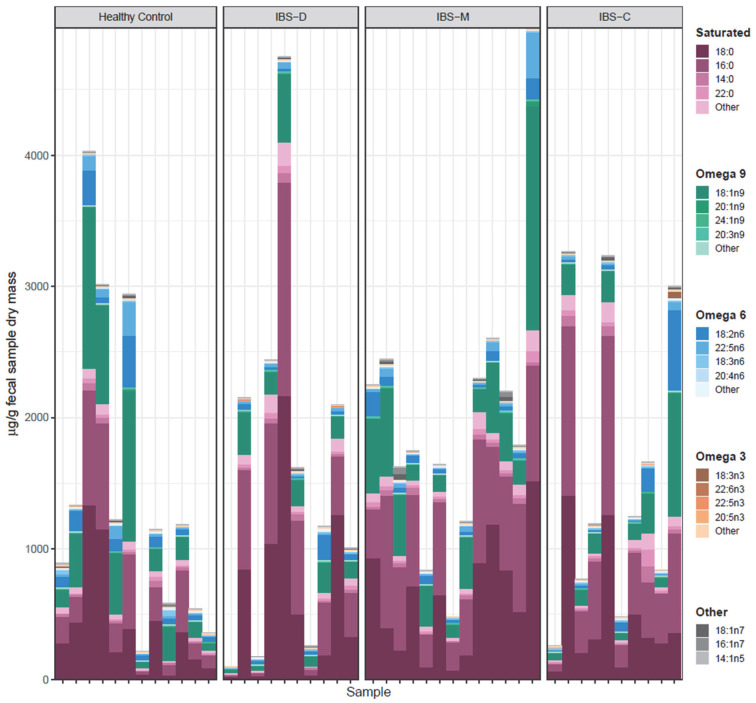
Stacked lipids bar plot of healthy controls versus IBS and IBS subtypes. Each bar represents one sample, and the total height of the bar represents the total concentration of all detected lipids. Colors representing each group (unsaturation position) of lipids are indicated as saturated (purple), omega 9 (green), omega 6 (blue), omega 3 (orange), and other (gray). Abbreviations: IBS, irritable bowel syndrome; IBS-D, irritable bowel syndrome–diarrhea; IBS-M, irritable bowel syndrome–mixed; IBS-C, irritable bowel syndrome–constipation.

**Figure 7:**
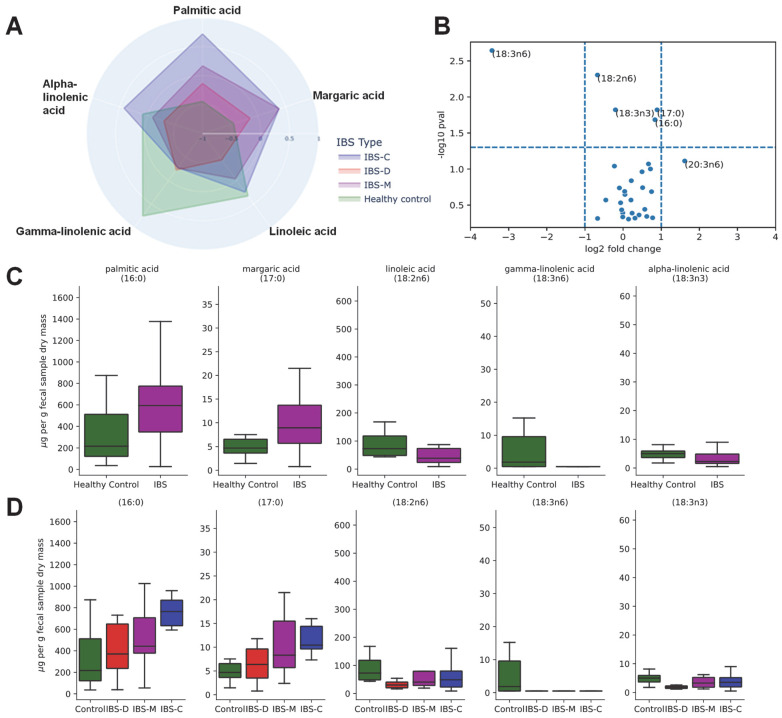
Lipidome analyses of healthy controls versus IBS and IBS subtypes. Transformed concentrations of lipids that were significantly different (per Kruskal–Wallis test) across healthy control participants and various IBS subtypes are presented via a spider plot (**A**). Each lipid’s concentrations were *Z*-score transformed, and the mean *Z*-score for each IBS subtype is plotted on each axis. Lipids that were significantly different (per Mann–Whitney U test) between healthy control participants and all IBS patients are presented in a volcano plot (**B**). Each point represents one lipid, the x-axis represents log-fold change from healthy controls to participants with IBS, and the y-axis represents the negative log of the *p* value. Points above the horizontal dashed line had a *p* < 0.05, and points outside of the vertical dashed lines had greater than a 2-fold change. Concentrations of significantly differing lipids, separated by IBS status and IBS subtype, respectively, are presented as boxplots with overlaid scatterplots in (**C**,**D**), respectively. Individual points represent the concentration of the lipid in one sample. Horizontal lines represent the median. Boxes indicate the 1st through 3rd quartiles of the lipid’s concentration in its IBS/subtype group, and whiskers represent the range up to 1.5× the interquartile range. Abbreviations: IBS, irritable bowel syndrome; IBS-D, irritable bowel syndrome–diarrhea; IBS-M, irritable bowel syndrome–mixed; IBS-C, irritable bowel syndrome–constipation.

To evaluate the impact of IBS on fatty acid composition, we performed a targeted lipidomics analysis of a panel of 30 fatty acids. These fatty acids included long-chain fatty acids (LCFAs), monounsaturated fatty acids (MUFAs), and polyunsaturated fatty acids (PUFAs). The stacked bar plot in Figure 6 shows the concentrations of detected lipids in fecal samples. Saturated fatty acids were predominant in all groups of samples. Please note that saturated fatty acids typically exist in higher concentrations, but unsaturated fatty acids can be highly bioactive in low concentrations. Concentrations of multiple lipids were significantly different (per Kruskal–Wallis test) across healthy control participants and various IBS subtypes (Figure 7A). A spider chart (also known as a radar plot) shows these lipids in Figure 7A, where each lipid has its own axis; all axes are joined in the center of the figure. Each lipid’s concentrations were *Z*-score transformed, and the mean *Z*-score for each IBS subtype is plotted on each axis. Mean *Z*-scores of significantly different lipids are shown for each group. Gamma linolenic acid (18:3n6) was significantly higher in the control participants than in IBS patients. Palmitic acid (16:0) was highest in the IBS-C patients, while margaric acid (17:0) was higher in the participants with IBS-M and IBS-C. Lipids that were significantly different (per Mann–Whitney U test) between healthy control participants and all IBS patients are presented in a volcano plot in Figure 7B. A positive log-fold change indicates higher concentration in the IBS patient group. Gamma linolenic acid (18:3n6) showed a level more than 2-fold higher in control participants than in IBS patients. Based on uncorrected Mann–Whitney U tests (*p* value < 0.05), certain saturated fatty acids (palmitic acid, 16:0; margaric acid, 17:0) were higher in IBS patients, while certain unsaturated fatty acids (linoleic acid,18:2n6; gamma-linolenic acid, 18:3n6; alpha-linolenic acid, 18:3n3) were lower in IBS patients. Gamma-linolenic acid (18:3n6) was the lipid with the largest fold change between groups. Concentrations of significantly differing lipids, separated by IBS status and IBS subtypes, respectively, are presented in boxplots with overlaid scatterplots in Figure 7C,D, respectively.

### 3.6. Link between the Gut Microbiome and the Fecal Lipidome Compositions via Procrustes Randomization Test

**Figure 8:**
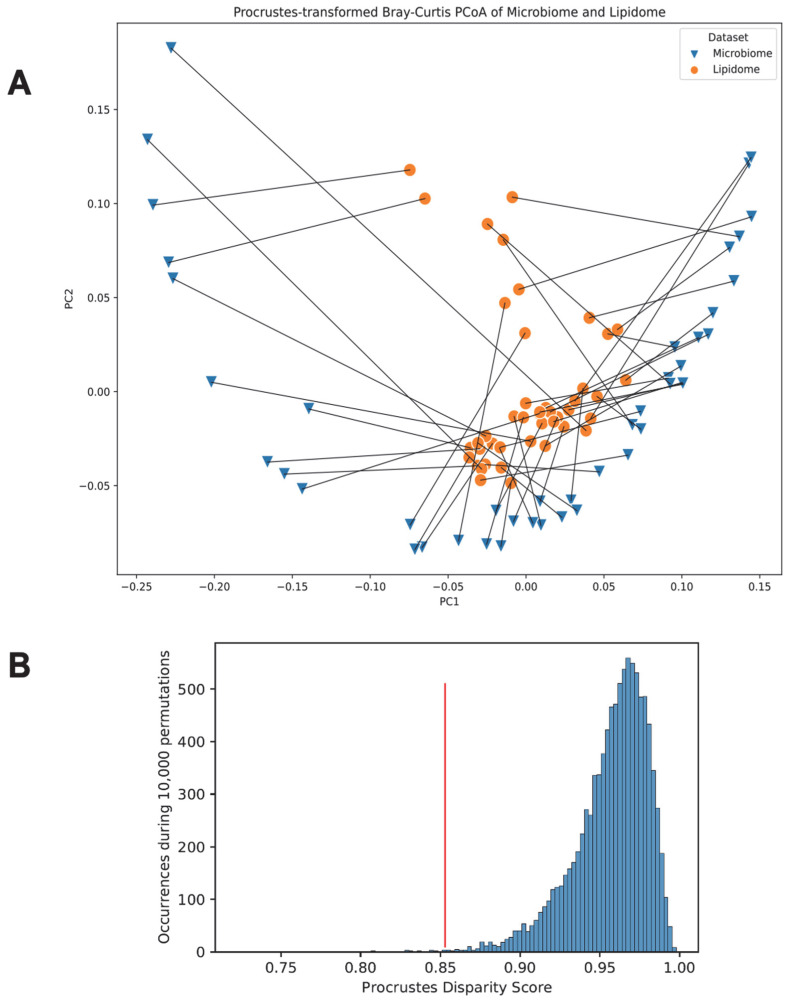
Procrustes-transformed Bray–Curtis multi-dimensional scaling of microbiome and lipidome. (**A**) Blue triangles indicate untransformed Bray–Curtis PCoA coordinates for the microbiome data, and orange circles indicate Procrustes-transformed Bray–Curtis PCoA coordinates of the lipidome. Each point indicates one participant’s sample, and lines are drawn between each participant’s microbiome and lipidome. (**B**) Histogram shows distribution of Procrustes disparity scores across 10,000 permutations of the dataset, and the vertical red line indicates the Procrustes disparity of our dataset. Abbreviations: PC, principal coordinates analysis axis; PCoA, principal coordinates analysis.

A Procrustes randomization test was carried out to explore the multivariate association between each participant’s microbiome and lipidome. As seen from Figure 8A, the microbiome and lipidome datasets associated much better than expected by random chance (empirical *p* = 0.002). A histogram showing the distribution of Procrustes disparity scores across 10,000 permutations of the dataset is presented in Figure 8B. The Procrustes disparity score for our data (shown by the red line in Figure 8B) was lower than 99.8% of disparity scores when we randomly sampled the data 10,000 times. This suggests that the microbiome and lipidome datasets fit together better than randomly matching the microbiome–lipidome samples 99.8% of the time.

### 3.7. Association of Socioeconomic Factors with Diversity and Community Composition of the Gut Microbiome

**Figure 9:**
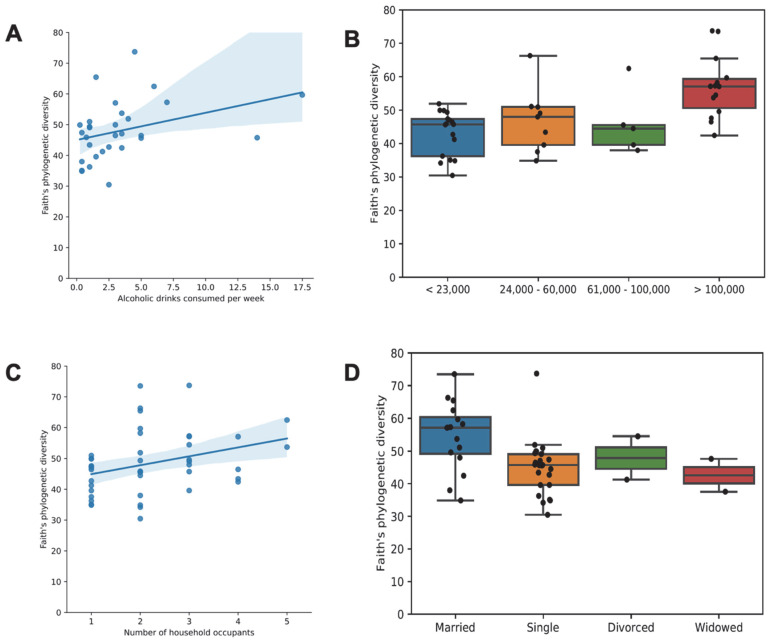
Faith’s phylogenetic alpha diversity of the microbiome as a function of socioeconomic factors. Panels (**A**,**C**) show the relationship between phylogenetic diversity and (**A**) alcohol consumption or (**C**) the number of household occupants. Each point represents one participant’s microbiome sample, the line represents the line of best fit, and the shaded region indicates the 95% confidence interval of a linear regression on Faith’s phylogenetic diversity as a function of alcohol consumption or the number of household occupants, respectively**.** Boxplots with overlaid scatterplots in panels (**B**,**D**) depict the relationship between phylogenetic diversity and (**B**) household income group or (**D**) marital status. Individual points represent the phylogenetic diversity of one participant’s sample. Horizontal lines represent the median. Boxes indicate the 1st through 3rd quartiles of the phylogenetic diversity of each group, and whiskers represent the range up to 1.5× the interquartile range.

**Figure 10:**
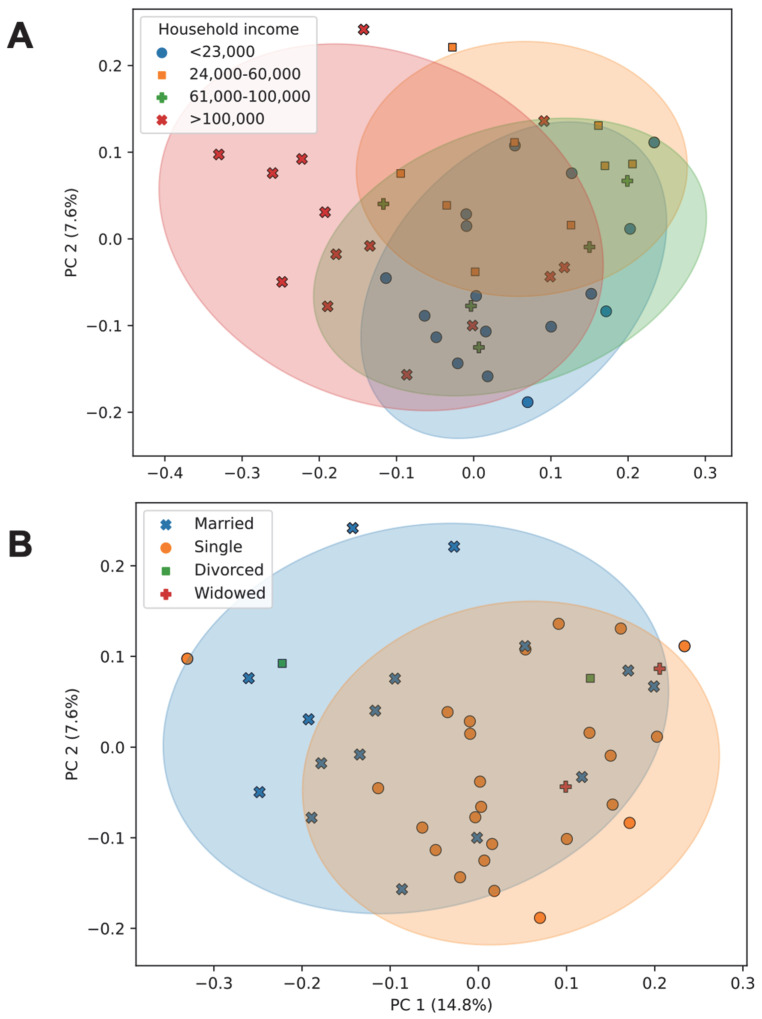
Microbiome beta diversity as a function of socioeconomic factors. (**A**) Unweighted UniFrac PCoA plots are shown comparing the phylogenetic composition of samples across household income groups and (**B**) marital status. Percentages along each axis show the portion of phylogenetic variance across samples captured by that axis. Each point represents the phylogenetic composition of one sample. Ellipses represent 95% confidence intervals of each group’s PCoA coordinates. Abbreviations: PC, principal coordinates analysis axis; PCoA, principal coordinates analysis.

As the manifestation of IBS symptomology is influenced by various socioeconomic factors, we also explored the influence of (i) alcohol consumption, (ii) household income, (iii) number of household occupants, and (iv) marital status using Faith’s phylogenetic alpha diversity analysis. We observed that a higher alcohol consumption was associated with a higher alpha diversity (Figure 9A). A higher income was associated with a higher alpha diversity, while a lower income corresponded to a lower alpha diversity (Figure 9B). An increased number of occupants (Figure 9C) and married status (Figure 9D) were associated with a higher alpha diversity. Additionally, household income (Figure 10A; pseudo-*F* = 1.64, *p* < 0.001) and marital status (Figure 10B; pseudo-*F* = 1.32, *p* = 0.028) were associated with differences in beta diversity (as measured using unweighted UniFrac).

## 4. Discussion

ANCOM, our network module method, and LEfSe all showed that the *Lachnospiraceae* group ND3007 was lower in participants with IBS than in healthy controls. In many studies, identified differentially abundant taxa are not robust across various differential abundance methods, so this finding should be particularly considered robust due to the consensus across differential abundance methods. Members of the Lachnospiraceae ND3007 group are short-chain fatty acid (SCFA) producers and have been linked to decreases in the blood glucose levels in rats with diabetes [49,50]. In general, bacteria belonging to the family Lachnospiraceae are considered important butyrate producers. Butyrate and other short-chain fatty acids are shown to modify gut motility, maintain the integrity of the intestinal barrier, and also play a role in the inhibition of intestinal inflammation [51]. SCFAs are essential in maintaining a homeostatic immune environment, and butyrate particularly has anti-inflammatory and immunoregulatory effects [52]. Additionally, butyrate stimulates serotonin release via enterochromaffin cells, altering sensory neuron activity, which is disrupted in individuals with IBS [53,54]. It has also been discussed that environmental and psychological stressors reduce the proportion of Lachnospiraceae. This may, in turn, lead to a reduction in mucus and SCFA production, as well as reductions in tight junction protein expression resulting in intestinal inflammation [55]. Our observation that the Lachnospiraceae group ND3007 was abundant in healthy control participants and was less abundant in IBS, which has inflammatory pathophysiology in many cases, is consistent with these findings.

It is likely that dietary differences in individuals with and without IBS may impact the relative abundance of Lachnospiraceae. Ma et al. reported that there is an association between diet quality and the relative abundance of Lachnospiraceae; specifically, persons with a higher healthy eating index showed higher levels of Lachnospiraceae ND3007 and Ruminococcaceae [56]. Other studies also showed that the dietary inclusion of fiber, antioxidants, and phytochemicals, for example, through the consumption of flaxseed and grapes, led to higher relative abundances of Lachnospiraceae ND3007 [57,58]. Moreover, it is evident that HFCS in the diet may alter the relative abundance of particular taxa [59,60,61]. A study showed a modest decrease in Ruminococcaceae with a high HFCS intake in an adolescent mouse model [62]. The decrease in Ruminococcaceae observed in the IBS patients in our study may, thus, at least partly be due to the high consumption of HFCS in these patients.

ANCOM also showed that *Eisenbergiella* was higher in IBS patients, especially those with IBS-C and, to a certain extent, IBS-M, relative to healthy controls. Species of the genus *Eisenbergiella* have been associated with various disease conditions such as increased risk of bacteremia, colorectal tumorigenesis, and multiple sclerosis [63,64,65]. To the best of our knowledge, our study is the first report of an alteration of genus *Eisenbergiella* in IBS patients, while higher levels of *Eisenbergiella* in the IBS group than in the control group have been reported in a mice model in one study [66]. The exact mechanism underlying these observations is not known, but it has been suggested that certain bacteria including *Eisenbergiella* may be associated with inflammatory factors [63]. Higher levels of *Eisenbergiella* spp. were also observed in persons following a high-saturated-fatty-acid diet, which often co-occurs in foods with HFCS [67]. Consistent with the effects of dietary saturated fatty acids on *Eisenbergiella*, our participants with IBS (particularly IBS-C) had high fecal concentrations of saturated fatty acids, including palmitic and margaric acids. This is also consistent with a recent study demonstrating that individuals with IBS have higher fecal concentrations of palmitic and margaric acids [68]. Bhat et al. also reported that short-term HFCS consumption in 6-week-old female mice increased circulating concentrations of palmitic acid, despite constant dietary palmitic acid consumption [62]. This suggests that though HFCS and saturated fatty acids often co-occur in foods, HFCS consumption may alter lipid metabolism and could be another mechanism explaining the higher fecal concentration of palmitic acid in IBS patients. Lipidome analysis of our fecal samples showed lower concentrations of fecal gamma linolenic acid in all subtypes of IBS patients than in healthy controls. Gamma linolenic acid has anti-inflammatory properties in multiple human tissues, and there could be multiple reasons for its lower concentration in IBS patients, including dietary differences, microbial metabolism of gamma linolenic acid/its precursors, or changes in enterocyte absorption/metabolism [69,70,71].

A study using a rat model showed that ethanol consumption led to a significant decline in the diversity of the gut microbiome. The authors compared their results to human fecal microbiome data collected by the American Gut Project. In contrast to the rat model data, human subjects who consumed alcohol had significantly higher gut microbial biodiversity than non-drinkers, ref. [72], consistent with other studies [73]. The authors dedicated this difference to a lack of prior ethanol exposure in rats. A recent study showed that the consumption of ethanol in the form of beer increased gut microbiome diversity, suggesting that beer may exert effects on the gut microbiome through polyphenols, rather than alcohol [74]. Thus, there is some contradicting evidence on the effects of alcohol on the gut microbiome across murine models and observational studies in humans, related to factors such as alcohol type and novelty of alcohol consumption. However, it is posited that the effects of alcohol on diversity of the gut microbiome could be mediated by polyphenols in drinks or by the sharing of microbes from social interactions during alcohol consumption.

Silvernale et al. showed that psychological comorbidities in IBS patients were associated with lower socioeconomic status and lower average per capita income [75]. A study showed that the risk of IBS was increased among unmarried participants [76]. Dill-McFarland observed that married individuals living together exhibited greater gut microbiome richness than individuals living alone [77]. These results support that sustained human interactions influence the gut microbiome. Wilmes et al. highlighted the potential for therapeutic targeting of the gut microbiome as a valuable strategy for the management of comorbid psychiatric symptoms in IBS [78].

Our sample size for this pilot study is modest, which is a limitation. This limitation was posed by the socioeconomic characteristics prominent in our patient community. Our local community faces significant economic challenges, and over 25% of the adults have not attained a high school diploma. Language is another barrier that was encountered during recruitment. It is possible that all these factors influence their appreciation of the relevance of the study, leading to less eagerness to participate. Logistic challenges such as lack of transportation or not being able to take time off from work or domestic responsibilities affected sample delivery to the clinic. Our recruitment was also hindered due to the COVID-19 pandemic and its aftereffects. The recruitment and sample collection started in 2019. Recruitment was stopped after the onset of the pandemic in March 2020 as the hospital switched to the telemedicine mode of patient care for ~3 months, and that, too, was used for emergency cases. Thus, patients that formed the basis of this study were not coming in for in-person, routine check-in visits for quite some time. Secondly, we did not recruit patients until COVID-19 tests were routinely used to rule out the presence of viral infection. This precaution was taken to avoid the influence of compounding factors. The stress created by the pandemic further diminished the enthusiasm among our participants to participate in this study, resulting in the current sample size. As our sample size was somewhat limited, we cannot rule out the possibility that certain conclusions will need further confirmation using a larger sample size.

However, despite all these challenges, we did find a number of important and meaningful observations, which led to new insights into IBS pathophysiology. Our robust statistical analyses using a number of different approaches, the significant statistical differences observed in the IBS patient and healthy control cohorts, and the consistency of our key observations with the reported literature from a number of different groups supported the validity of the outcomes. As mentioned above, our study underscored the influence of HFCS consumption and socioeconomic factors in IBS pathophysiology based on distinct microbiome and lipidome differences. Observations from this study may thus inform IBS patient care.

## Figures and Tables

**Table 1:** Patient demographics and clinical characteristics.

Characteristics	Number of IBS Patients	Number of Healthy Control Participants
	***n* = 33(%)**	***n* = 12(%)**
**Gender**		
F	26(78.8)	6(50.0)
M	7(21.2)	6(50.0)
**Age**		
18–39	12(36.4)	6(50.0)
40–80	21(63.6)	6(50.0)
**Race**		
African American	2(6.1)	1(8.3)
Hispanic	1(3)	0(0)
Caucasian	28(84.8)	9(75%)
Asian	2(6.1)	2(16.7)
**Marital Status**		
Widowed	2(6.1)	0(0)
Single	15(45.5)	10(83.3)
Separated/Divorced	2(6.1)	0(0)
Married	14(42.4)	2(16.7)
**IBS Subtype**		
IBS-Diarrhea	10(30.3)	None
IBS-Constipation	10(30.3)	None
IBS-Mixed	13(39.4)	None
**Hypertension**		
Yes	10(30.3)	1(8.33)
No	23(69.7)	11(91.7)
**Diabetes**		
Yes	3(9.1)	0(0)
No	30(90.1)	12(100)
**High cholesterol**		
Yes	6(18.2)	0(0)
No	27(81.8)	12(100)
**Average BMI**	27.5	23.14
**Depression**		
Yes	12(36.4)	0(0)
No	21(63.6)	12(100)
**Anxiety**		
Yes	13(39.4)	0(0)
No	20(60.6)	12(100)
**PTSD**		
Yes	2(6.1)	0(0)
No	31(93.9)	12(100)
**Bipolar**		
Yes	2(6.1)	0(0)
No	31(93.9)	12(100)
**Psychosis**		
Yes	0(0)	0(0)
No	31(93.9)	12(100)
**Smoking**		
Yes	1(3)	0(0)
No	32(97)	12(100)
**Alcohol**		
Yes	18(54.5)	12(100)
No	15(45.5)	0(0)

## Data Availability

Code and data for reproducing these analyses can be accessed at https://github.com/sterrettJD/Phadtare-IBS. Raw sequencing data are available on request from the corresponding author.

## Supplementary Materials

The following supporting information can be downloaded at: https://www.mdpi.com/article/10.3390/microorganisms11102503/s1, Table S1. Detailed composition of modules.
